# The COVID‐19 pandemic reduced the risk of febrile neutropenia during inpatient chemotherapy for urological cancer

**DOI:** 10.1111/cas.15490

**Published:** 2022-07-15

**Authors:** Ren Toriumi, Hiroshi Yaegashi, Suguru Kadomoto, Hiroaki Iwamoto, Masashi Iijima, Shohei Kawaguchi, Takahiro Nohara, Kazuyoshi Shigehara, Kouji Izumi, Yoshifumi Kadono, Atsushi Mizokami

**Affiliations:** ^1^ Department of Integrative Cancer Therapy and Urology Kanazawa University Graduate School of Medical Science Kanazawa Ishikawa Japan

**Keywords:** COVID‐19, Febrile Neutropenia, Drug Therapy, Hygiene, Fever of Unknown Origin

## Abstract

Since 2020, the COVID‐19 pandemic has led to the widespread practice of hand hygiene and wearing face masks, not only among healthcare workers, but also among the general public. Thus, there is a need to verify the impact of the COVID‐19 pandemic on the incidence of febrile neutropenia (FN). This study aimed to examine the incidence of FN in hospitalized chemotherapy patients at Kanazawa University Hospital. Among inpatients at the Department of Urology receiving chemotherapy, we compared the incidence of FN between 317 cases in 2018–2019 and 276 cases in 2020. We retrospectively analyzed the factors of FN via binominal logistic regression analysis based on patient characteristics and the characteristics of primary diseases, with statistical significance set at *p* < 0.05. FN occurred in 20/317 cases in 2018–2019, but only in 1/276 cases in 2020, with a significant decrease in the latter (*p* = 0.005). On multivariate analysis, we identified the following independent risk factors for FN: non‐COVID‐19 era (*p* = 0.005), first course of therapy (*p* = 0.005), malnutrition (*p* = 0.032), and past history of FN (*p* = 0.018). Due to the COVID‐19 pandemic, hygiene policies for healthcare workers and quarantine measures for patients were thoroughly implemented. As a result, the incidence of FN in 2020 decreased to 1/15 of the previous incidence. Thus, hygiene measures for healthcare workers and patients during the expected period of chemotherapy‐induced neutropenia are important for FN prevention.

